# Chirped Laser Dispersion Spectroscopy for Remote Open-Path Trace-Gas Sensing

**DOI:** 10.3390/s121216466

**Published:** 2012-11-28

**Authors:** Michal Nikodem, Gerard Wysocki

**Affiliations:** Electrical Engineering Department, Princeton University, Princeton, NJ 08540, USA

**Keywords:** optical dispersion spectroscopy, laser spectroscopy, remote sensing, quantum cascade lasers, PACS42.62.Fi (Laser spectroscopy), 42.68.Wt (Remote sensing)

## Abstract

In this paper we present a prototype instrument for remote open-path detection of nitrous oxide. The sensor is based on a 4.53 μm quantum cascade laser and uses the chirped laser dispersion spectroscopy (CLaDS) technique for molecular concentration measurements. To the best of our knowledge this is the first demonstration of open-path laser-based trace-gas detection using a molecular dispersion measurement. The prototype sensor achieves a detection limit down to the single-ppbv level and exhibits excellent stability and robustness. The instrument characterization, field deployment performance, and the advantages of applying dispersion sensing to sensitive trace-gas detection in a remote open-path configuration are presented.

## 1. Introduction

Laser technology offers unique remote non-invasive chemical sensing capabilities, and plays an important role in a broad range of applications such as industrial monitoring and emission control [1,2] exhaled breath analysis for medical diagnostics [3,4], or security and safety applications [5]. One of the most dynamically developing areas of laser-based spectroscopic sensing is focused on environmental monitoring. Despite the environmental importance of various atmospheric trace-gases (CO_2_, N_2_O, NO, CH_4_, O_3_, *etc.*), exact knowledge about the location and magnitude of their emission sources and sinks is still very limited. In this respect, remote sensing technologies can offer a convenient and cost-effective solution for field deployed sensors that can monitor large areas while providing good temporal and spatial resolution of precise trace-gas concentration data.

Most of the currently available remote sensors are based on optical detection that utilizes measurements of light absorption to determine chemical composition of the sample. At shorter wavelengths in the ultra-violet and visible spectral range, laser detection and ranging (LIDAR) technologies that rely on backscattering of light from particles in the atmosphere can also be used for chemical detection if spectroscopic information is being retrieved. Differential techniques such as differential absorption LIDAR (DIAL) utilize measurements at different optical frequencies for spectroscopic information retrieval, but the sensitivity, and most importantly, the specificity of those systems is moderate [6]. Particularly important for chemical sensing is the mid-infrared (mid-IR) spectral region, where most chemicals in the gas phase exhibit their strongest ro-vibrational absorption bands. However, scattering in the atmosphere is less effective at longer wavelengths and remote sensing is usually implemented in a hard-target LIDAR configuration, often with retroreflectors used as hard targets to enhance return optical signals. A majority of remote sensing spectroscopic LIDAR/DIAL systems use a direct laser absorption spectroscopy (DLAS) technique or its derivatives based on wavelength modulation spectroscopy (WMS). In DLAS the measured signal is typically detected as a small intensity change over a large background of the total transmitted laser intensity. Due to transmission variations, which are likely to occur in remote sensing configurations, the DLAS baseline is prone to significant fluctuations that might be misinterpreted as a molecular absorption. WMS is known to suppress baseline and 1/f noise and thus can improve detection sensitivity. However, the WMS signal is still intensity-dependent and requires normalization by the received power, which especially in low-light conditions might be a source of a significant measurement error. Such a power normalization using the first harmonic of the WMS signal, or through direct power level monitoring, was successfully demonstrated in the near-infrared spectral region (near-IR) where excellent photodetectors with negligible nonlinearities are available [7,8]. In the mid-infrared (mid-IR) photodetectors are usually strongly nonlinear, which makes such normalization far more challenging or impossible. Therefore there is a clear need for remote sensing technologies that could mitigate those limitations of DLAS/WMS while offering cost effective solutions for remote chemical sensing over large areas with high sensitivity and chemical selectivity.

Recently we have introduced the concept of chirped laser dispersion spectroscopy (CLaDS), a novel technique for quantitative trace gas detection based on molecular dispersion measurements [9]. In contrast to most gas sensing methods, CLaDS does not measure absorption but instead it detects refractive index changes that occur in the vicinity of a molecular transition. Because CLaDS is essentially a phase detection technique, the CLaDS measurement is highly immune to variations of optical power at the photodetector. This makes it particularly suitable for remote open-path sensing, where the received power level may strongly fluctuate. In this paper a CLaDS-based system developed for open-path remote sensing of nitrous oxide (N_2_O) is discussed. A complete system characterization and demonstration of open-path N_2_O sensing, both in the laboratory and in the field, have been performed.

## 2. CLaDS Basic Operation Principles

When the frequency of an electromagnetic wave coincides with a resonance of the irradiated medium (e.g., rotational, vibrational or electronic) absorption and dispersion of the transmitted light wave occurs. The amount of dispersion (measured as a change in refractive index per unit frequency) is proportional to the concentration of the absorbing species, thus it can be used for quantitative concentration measurements. Attempts to use refractive index measurements for molecular detection had already been made in 1902 [10], but only a few techniques have been implemented to-date for trace gas detection [11–13], and none of them is suitable for remote sensing. In contrast, the ClaDS technique is a promising dispersion-based method that can be used in a remote-sensing configuration and may become an attractive alternative to absorption-based techniques that rely on the Beer-Lambert law.

**Figure 1.**
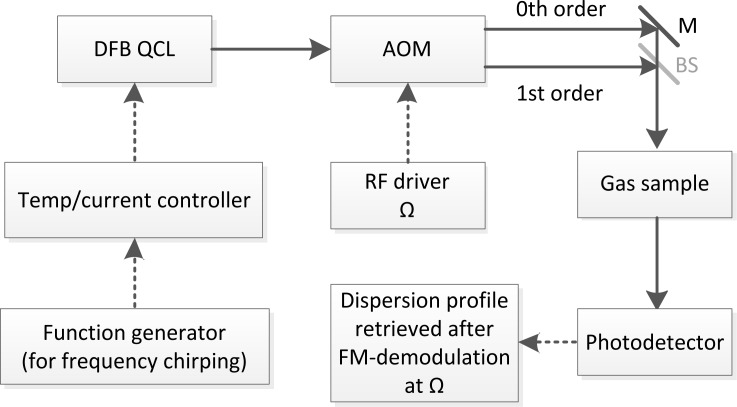
Schematic diagram of mid-IR CLaDS setup (M-mirror, BS-beam splitter).

The theory of CLaDS has been discussed in detail in Refs. [9,14]. Here we provide only the necessary details needed for a self-contained description. A schematic diagram of the mid-IR CLaDS setup based on a distributed feedback (DFB) quantum cascade laser (QCL) is shown in Figure 1. In general, the output radiation from a frequency-chirped single-mode laser is directed through an optical frequency shifter (e.g., acousto-optical modulator-AOM). Electrically pumped semiconductor laser sources, especially QCLs operating in the mid-IR, are particularly suitable for this application due to their flexibility and convenience in terms of laser frequency modulation and control. However, any other single-mode laser source that allows for controlled frequency chirping can be used in CLaDS. Both the fundamental and the frequency shifted wave are combined into a single dual-frequency laser beam (in case of the AOM shifter the Mach-Zehnder interferometer arrangement is used). The dual-frequency beam is sent through a gas sample and is focused onto a fast photodetector that extracts the heterodyne beat-note between both frequency components. When the dual-frequency beam interacts with a molecular transition both wavelengths experience slightly different refractive indices. As the laser is frequency-chirped, the difference in propagation velocities impacts the instantaneous frequency difference between the two optical waves, and this effect (that is proportional to the chirp rate) can be directly measured as an instantaneous frequency of the heterodyne beat-note signal. This signal enhancement due to the frequency chirp rate is central to the concept of CLaDS signal generation [9]. The dispersion spectrum is obtained through frequency demodulation of the heterodyne beat-note and the molecular concentration can be retrieved through spectroscopic modeling based on parameters available from spectral databases or by using prior calibration of the system with reference gases.

In this basic arrangement (as shown in Figure 1) by applying appropriate modulation to the laser injection current a linear chirp across the target molecular transition can be obtained and the dispersion spectrum is directly recorded using FM-demodulation of the heterodyne signal [9]. Such a configuration and data processing is defined as direct CLaDS. The most significant advantage of the CLaDS technique over a conventional absorption based measurement is the immunity of the molecular dispersion measurement (performed in the frequency domain) to fluctuations of the received optical power. As a consequence, the large baseline that is typically observed in DLAS is not present in CLaDS. Due to exponential character of the Beer-Lambert law, the non-linear dependence of the absorption on sample concentration represents another important drawback of DLAS, especially for samples with absorption larger than 10%. In contrast, the dispersion signal increases linearly with concentration, resulting in an extended dynamic range for concentration measurements [15] that also allows for longer optical distances to be used in path-integrated molecular studies. The latter can significantly benefit remote sensing applications that rely on multiple optical paths of different lengths to perform tomographic measurements of atmospheric constituents [16,17]. Despite the fundamental advantages of direct CLaDS, previous studies have revealed some limitations in this technique. Although theory suggests that the CLaDS signal is baseline-free, in practice a weak baseline can be observed when the interferometer in the direct CLaDS setup is not perfectly balanced and the chirp rate is not truly linear (both can occur in practical implementations) [9]. Moreover, with chirp rates typically between 10^12^ and 10^15^ Hz/s, the acquisition bandwidth (BW) required to capture the dispersion spectrum must be very broadband, which leads to an increase in the FM-demodulation noise (proportional to BW^2^). On the other hand, with lower chirp rates the CLaDS signal amplitude is proportionally reduced and the signal-to-noise ratio (SNR) decreases [18]. Hence, direct CLaDS requires the chirp rate and acquisition bandwidth to be carefully optimized for the best performance.

In order to overcome the identified limitations in direct CLaDS, a CM-CLaDS technique that is based on a harmonic detection scheme has been developed [19]. In the QCL-based CM-CLaDS the laser frequency is chirped with a fast sinusoidal waveform added to the injection current. The heterodyne detection followed by the FM-demodulation is similar as in direct CLaDS. However, the CM-CLaDS signal is recovered in the post-processing of the time series of the FM-demodulated beat-note frequency. The beat-note frequency time-series undergoes Fourier analysis and the amplitude of the 2nd harmonic component of the chirp modulation frequency is recorded as the CM-CLaDS signal. Narrowband analysis of the 2f signal allows reduction of the acquisition bandwidth, which leads to reduction of the FM-noise and increases the SNR. Moreover, it has been shown in [19] that in CM-CLaDS only the first harmonic is affected by the imbalance of the interferometer arms. Therefore, the 2nd harmonic signal is baseline-free which allows for convenient operation of CM-CLaDS system in a line-locked mode (laser frequency is locked to the peak of the transition) without the need for laser frequency scanning and spectroscopic data fitting for concentration retrieval.

A transition from direct CLaDS to CM-CLaDS is analogous to the transition from DLAS to WMS that also allows for continuous monitoring of the sample concentration in a line-locked mode. However, in WMS the 2f signal must be corrected for the laser power/sample transmission fluctuations [20–22]. Such corrections might be challenging or even not possible in the presence of large changes in the received optical power levels, especially when using mid-infrared photodetectors that usually have nonlinear response. Moreover, such nonlinearities can create a baseline in the WMS spectrum that generates additional errors in 2f/1f normalization. These issues are not affecting CM-CLaDS signal due to its immunity to optical power variations [19].

## 3. Remote Sensing with CM-CLaDS

**Figure 2.**
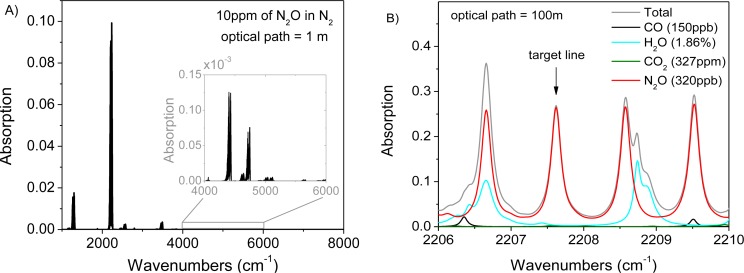
(**A**) A HITRAN simulation showing that the N_2_O absorption band near 2,200 cm^−1^ is significantly stronger than absorption bands at other wavelengths (inset shows magnification of the near-IR transitions); (**B**) A simulated absorption spectra for four atmospheric molecules shows that the transition at 2,207.6 cm^−1^ is optimal for N_2_O detection with minimum interference from water vapor, carbon monoxide, and carbon dioxide.

A preliminary test of remote open-path sensing with CM-CLaDS was performed using nitrous oxide as the target molecule. N_2_O is an important greenhouse gas and atmospheric pollutant [23]. Agricultural emissions are the primary source of a gradual increase of N_2_O concentration in the atmosphere at almost 1 ppbv/year rate [24,25]. Unfortunately, the global budget of N_2_O is still poorly characterized. In order to analyze changes in atmospheric N_2_O concentration, an instrument that provides precision and accuracy at a single ppbv level is required. Such performance can be achieved with mid-IR QCLs that give access to the strongest N_2_O transitions around 2,200 cm^−1^ (Figure 2(A)) [26,27]. The N_2_O P18e transition located at 2,207.62 cm^−1^ exhibits very small interference from other atmospheric constituents (such as H_2_O or CO) which makes it ideal for sensing in ambient conditions (Figure 2(B)).

**Figure 3.**
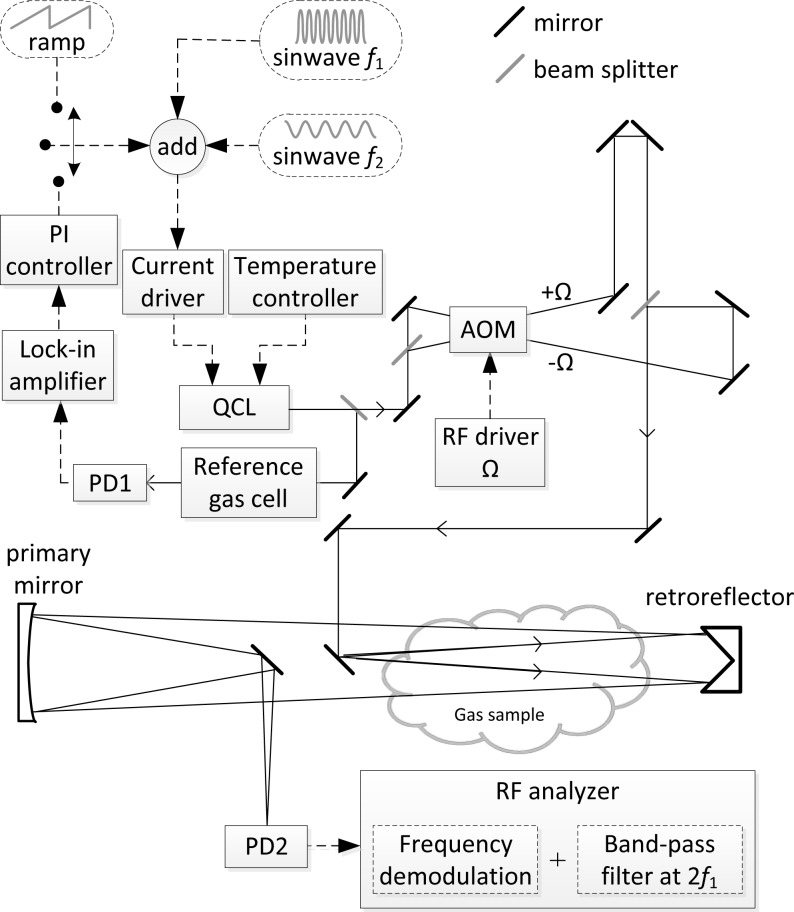
Optical setup of CM-CLaDS sensor for remote sensing (PD-photodetector).

The experimental arrangement of the prototype CM-CLaDS sensor for remote N_2_O detection is shown schematically in Figure 3. It was configured as a transportable system with optics placed on a 24″ × 36″ breadboard and installed with all the necessary electronics on a cart. The total power consumption of this transportable platform is ∼500 W (primarily limited by an off-the-shelf RF spectrum analyzer consuming ∼300 W). A thermoelectrically cooled (TEC) DFB QCL (provided by Corning Inc., Corning, NY, USA) generated up to 5 mW of optical power at 4.53 μm. By changing the laser temperature and bias current multiple N_2_O transitions between 2,207.6 and 2,211.4 cm^−1^ could be accessed. A CaF_2_ wedge plate was used to out-couple a small fraction of the laser power for active laser frequency locking. The main laser beam was divided with 50/50 beam splitter and the two beams were directed into the AOM. Both incident angles on the AOM were set to provide two Bragg reflected beams with one of them being up-shifted and the second down-shifted in frequency by Ω = 50 MHz. Both beams were re-combined into one beam using the Mach-Zehnder arrangement.

The laser power available for measurement of N_2_O at 2,207.62 cm^−1^ was only 250 μW due to losses on multiple optical components placed after the laser (AOM, beam splitters, several mirrors), but this was still sufficient to achieve a satisfactory SNR. This power was directed towards a distant retroreflector (diameter of 65 mm). Due to the divergence of the laser beam, a 6 inch concave mirror was used to collect the returning radiation and to focus it onto high-speed TEC MCT photodetector (PVI-3TE-10.6 from Vigo Systems S.A., Warsaw, Poland). The optical configuration of the collection optics can also be used as a standard Newtonian telescope to collect the scattered optical radiation from objects/hard-targets rather than a specular reflection from a retroreflector. The laser was frequency-chirped using a sinusoidal waveform at a modulation frequency of f_1_ = 101 kHz and with an optimized optical frequency modulation depth [19]. Due to imperfections of the antireflection coatings on the AOM facets the signal contained narrowband optical fringes created by an etalon formed between the AOM facets. In order to suppress these etalon fringes an additional small-amplitude modulation (at frequency f_2_ = 1 kHz) was applied to the laser current, which allowed suppression of the fringe pattern through averaging. The system could be operated in two modes: (1) a spectral measurement mode with a slow scan of the laser bias current to acquire a full 2f CM-CLaDS spectrum, or (2) a line-locked mode which enables continuous monitoring of N_2_O by CM-CLaDS. The latter was implemented using a proportional-integral (PI) controller to actively adjust the laser bias current using the 3f WMS as an error signal to keep the laser frequency at the center of the target transition. The heterodyne signal recorded by the photodetector was analyzed using an RF spectrum analyzer (RSA6106A from Tektronix, Beaverton, OR, USA). The time series of the beat-note frequency was digitally filtered in order to retrieve a 2f_1_ component that is proportional to the N_2_O concentration.

### 3.1. System Characterization

**Figure 4.**
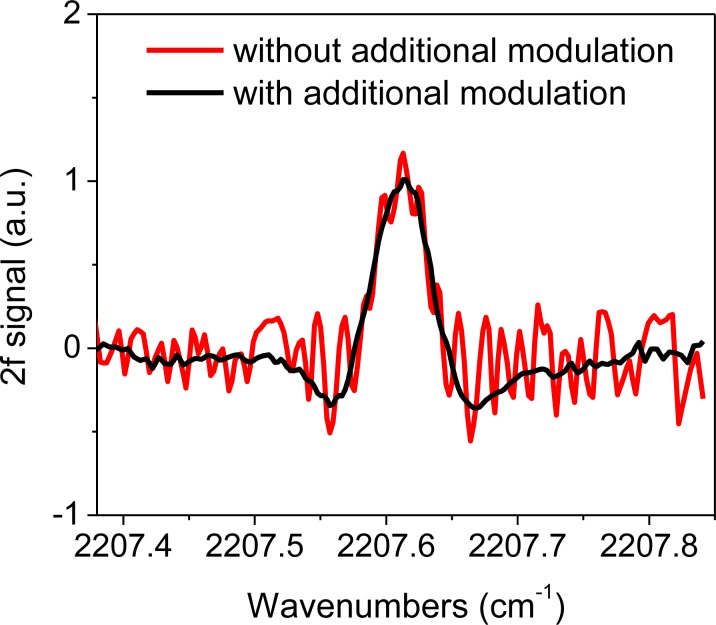
An example of the 2f CM-CLaDS spectrum recorded in the remote configuration with and without additional small-amplitude wavelength modulation at f_2_ (the sample is approximately 35 m of lab air at atmospheric conditions). The narrow fringes (red) are effectively suppressed with the modulation turned on (black).

Characterization of the CM-CLaDS instrument was conducted in the laboratory using two setups: a remote sensor arrangement with a retroreflector positioned ∼17 m away, or a setup with a 10 cm long gas cell filled with 151 ppmv N_2_O in N_2_ at 300 Torr pressure for calibration purposes. A significant improvement in CLaDS system performance was achieved through suppression of the parasitic optical fringes originating from the AOM. Because the length of the AOM frequency shifter is >5 cm (which for germanium corresponds to an optical length of >20 cm), the free spectral range of the observed parasitic fringes is much smaller than the line width of the target spectral feature (>0.1 cm^−1^). Hence, additional non-synchronized sinusoidal modulation was very effective at fringe suppression. The effect of the additional modulation of the laser current at frequency f_2_ is clearly visible in Figure 4, which compares N_2_O spectra acquired with and without the fringe suppression.

**Figure 5.**
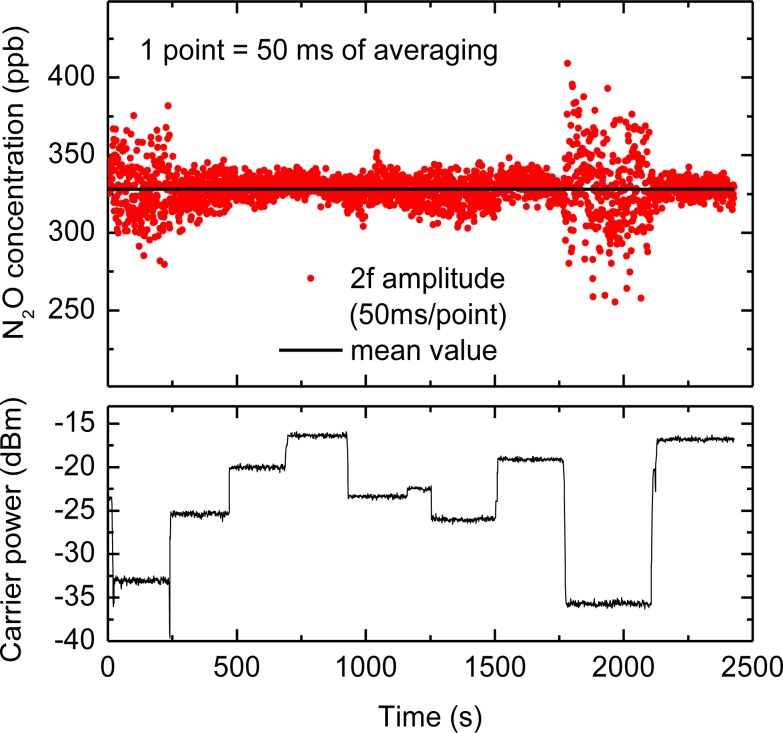
A continuous measurement of N_2_O concentration (upper plot) performed in the laboratory using an open-path configuration with a retroreflector placed ∼17 m away from the instrument. The carrier (beat-note) power (lower plot) is varied through changes of received optical power through partial obstruction of the telescope.

The CLaDS signal is strongly immune to changes in the received optical power [9,19]. This is especially important in open-path sensing over long distances when mechanical drifts in the system, turbulence in the atmosphere, or environmental conditions (dust, fog) can significantly affect the amount of power that is received by the photodetector. This property was discussed in detail for direct CLaDS in [9,18] and has been experimentally investigated in this work for CM-CLaDS by measuring the amplitude of 2f signal while the received power was varied. The laser wavelength was locked to the peak of the transition for continuous N_2_O detection, and the beat-note (carrier) level was varied through partial obstruction of the primary mirror. The concentration measurements and the variations of the RF signal power are shown in Figure 5. In FM-demodulation there is a slight dependence of the output noise on the carrier-to-noise ratio [28], which affects the SNR of the system. This is also observed in our instrument. Nevertheless, it is clear that despite power changes by more than 20 dB (100 times), the mean value of the CLaDS signal was not affected.

**Figure 6.**
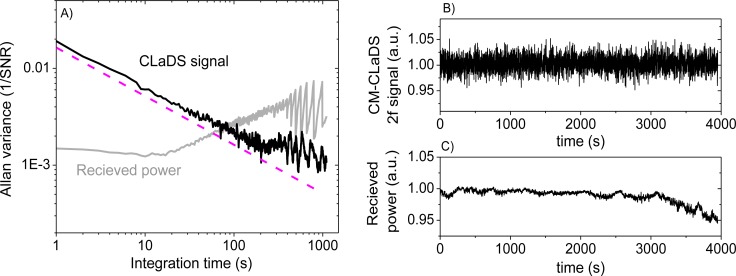
The Allan variance analysis (**A**) was performed for: (**B**) a time series of CM-CLaDS signal (normalized to the first data point) acquired for 151 ppmv N_2_O in N_2_ mixture in a 10 cm gas cell at 300 Torr, and (**C**) a received RF power series that was acquired simultaneously with data in (**B**). Active locking of the laser frequency to the peak of the transition at 2,207.6 cm^−1^ was enabled during this measurement. A dashed magenta line in (**A**) shows a theoretical performance of a random noise-limited system.

To test the stability of the CM-CLaDS system, an arrangement with a gas cell containing a calibrated gas mixture instead of a remote sensing setup was used. A gas mixture of 151 ppmv N_2_O in N_2_ was flowed through the cell with a fixed pressure of 300 Torr. Due to the limited data acquisition capabilities of the spectrum analyzer, an acquisition of a continuous data stream could not be performed. Hence, the signal was recorded for only 50 ms with an interval of 1 s between the consecutive acquisitions (5% duty cycle; the remaining 950 ms in the cycle were used for data processing). This has resulted in a 20-fold reduction in the effective integration time, but at the same time it enabled long-term stability analysis with a quasi-continuous signal acquisition. Both the CM-CLaDS signal as well as the RF beat-note power were simultaneously recorded and are shown in Figure 6. Allan variance plots were calculated for both data series. The received laser power has changed by more than 5% during the measurement (mainly due to line-locking feedback that adjusted the laser current to compensate for laser frequency drift caused by ambient temperature fluctuations). This drift is clearly visible in the Allan Variance calculated for the beat-note power in Figure 6. However, due to the CLaDS signal immunity to power variations, those drifts did not affect stability of the CM-CLaDS system. Based on the Allan plot the CM-CLaDS signal can be effectively integrated for up to one thousand seconds and it does not show a drift that is clearly observed in the Allan plot for the RF power (power drift starts dominating at integration times longer than 20 s). The ultimate SNR of 1,000 that is obtained for the maximum integration time tested (1,000 s) yields a minimum detection limit of 15 ppbv×m. It should be noted that the system performance in its current configuration is far from optimal because the frequency split achievable with commercially available AOMs (<100 MHz) is not sufficient to match the observed transition line width (∼3 GHz at atmospheric pressure). The signal loss due to non-optimal frequency splitting (100 MHz in this work) reduces the signal by ∼7 times with respect to an optimized signal at 300 Torr and by ∼16 times with respect to an optimized signal at atmospheric pressure. In addition to this signal loss, the 5% data acquisition duty cycle results in ∼ $\sqrt{20}$ times higher noise. Based on this stability test a bandwidth- and optical-path-normalized detection limit to N_2_O interpolated for conditions relevant for remote open-path atmospheric sensing is ∼1.2 ppmv×m/Hz^1/2^ (current system specifications have been used). This can be improved to below 50 ppbv×m/Hz^1/2^ with an optimum frequency shifter and a 100% duty cycle data acquisition. However, despite the performance limitations, the current system can still achieve the single-ppbv N_2_O detection limits required for atmospheric studies when implemented with longer (>50 m) optical paths.

### 3.2. Ambient N_2_O Sensing

**Figure 7.**
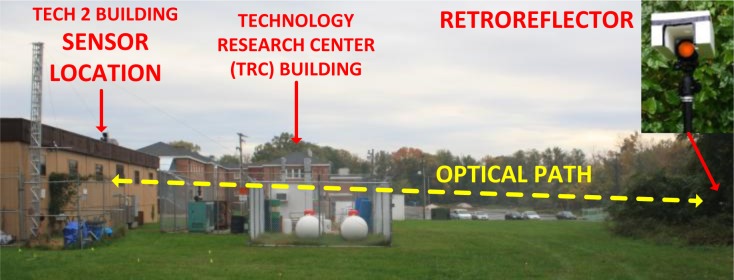
Picture of the environmental measurement site on the UMBC campus in Maryland. Locations of the sensor and the retroreflector are labeled.

To demonstrate capabilities suitable for environmental N_2_O monitoring, the CM-CLaDS prototype setup was deployed in a field environmental measurement site. The system was installed at a measurement site on the University of Maryland Baltimore County (UMBC) campus, about 8 km from Baltimore, MD, USA (Figure 7). The CM-CLaDS sensor was placed inside a non-air-conditioned building and was operated almost continuously for 5 days in October 2011 with only one significant interruption on 26 October due to a power outage. The laser beam was directed through an open window towards a distant retroreflector placed outside the building, yielding a total optical path of 71 m above a neighboring meadow.

**Figure 8.**
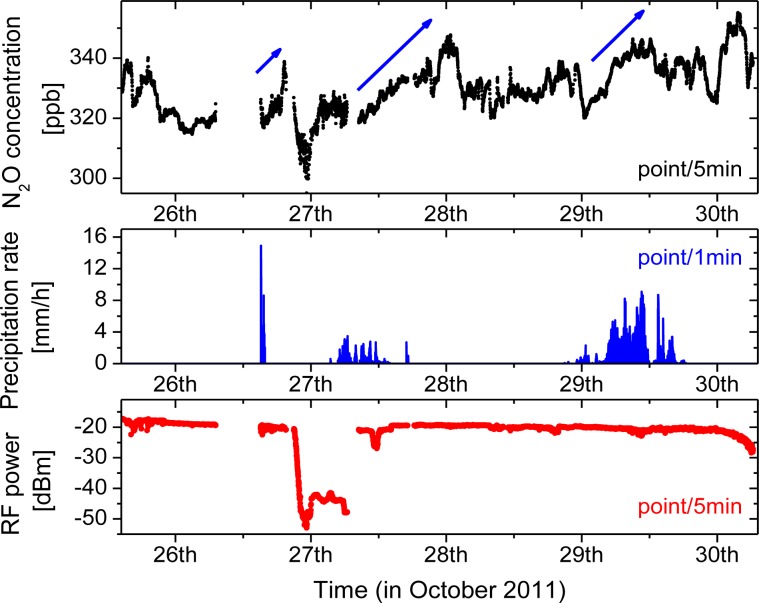
**Top plot:** A long-term atmospheric N_2_O concentration measurement during the field deployment of the CM-CLaDS sensor (5 min. average of data points recorded in 2 s intervals). **Bottom plot:** The corresponding heterodyne beat-note power indicates opto-mechanical drift of the setup and fluctuations in atmospheric transmission due to varying environmental conditions. **Center plot:** A precipitation rate recorded using an on-site weather station. Each rainfall event is followed by an increase in N_2_O concentration (indicated with arrows) which most likely is a result of nitrification and denitrification processes in soil.

The laser temperature and current were set to 25 °C and 150mA, respectively, which allowed targeting the optimal N_2_O transition at 2,207.62 cm^−1^. The signal processing was further optimized and enabled acquisition with a higher duty cycle of 12.5% (250 ms of data with 2 s intervals). The CLaDS signal was calibrated using a certified gas mixture composed of 151 ± 1 ppmv of N_2_O in dry N_2_ at pressure of 760 Torr, which determines the ultimate relative accuracy of concentration measurements to ±0.66% (approximately ±2.2 ppbv at the expected ambient conditions). The atmospheric N_2_O concentration time series was recorded and is shown in Figure 8.

Atmospheric temperature and pressure data were measured with a close-by weather station, and both data sets have been used to correct the measured molecular concentration based on the ideal gas law and spectroscopic parameters of the transition. A correction for water vapor in the atmosphere was also applied using the data from a local hygrometer. Each point (black dot) in Figure 8 is an average of data recoded within 5 min (150 points with 2 s interval and effective duty cycle of 12.5%).

The RF beat-note power was recorded simultaneously with the CM-CLaDS signal. Due to mechanical drift in the optical arrangement (temperature fluctuations *etc.*) during deployment, the beat-note power was slowly decreasing from −16 dBm on the first day down to slightly below −20 dBm by 30 October. There were also additional changes in the amount of received optical power caused by changing environmental conditions. The most significant occurred on 10/27 when the beat-note power dropped by more than 20 dB due to atmospheric water condensation on the retroreflector. The signal power was recovered after cleaning the retroreflector. Some other smaller power variations visible in the data were caused primarily by precipitation events.

Similar to the laboratory tests shown in Figures 5 and 6, the CM-CLaDS signal measured in the field also shows high immunity to changes in the received optical power. Continuous monitoring of N_2_O concentration was possible even in the event of >20 dB drop in the received power. Despite an increase in the noise, the mean value of the measured N_2_O concentration was not affected during this event (27 October morning *vs.* afternoon).

**Figure 9.**
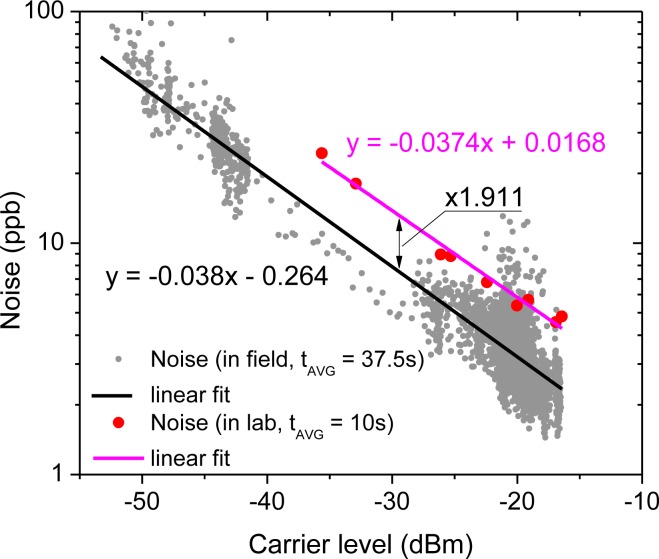
The CM-CLaDS N_2_O concentration data noise *vs.* carrier power level based on laboratory (red) and field (grey) measurements in Figures 5 and 8, respectively. For field data the noise is calculated as the standard deviation of 150 consecutive data points and each point corresponds to a 250 ms long data acquisition of the optical signal. This yields an effective averaging time of 37.5 s. For laboratory data from Figure 5 (measured with an effective averaging time of 10 s) the noise was calculated as standard deviation of 200 consecutive data points and each point corresponds to a 50 ms long data acquisition of optical signal. Both data sets were fitted by linear functions shown in black (for field data) and in magenta (for laboratory data).

As shown in Figure 9, the noise dependence on the received power level observed in the field (Figure 8) is in good agreement with the performance tested in the laboratory (based on data from Figure 5). In both cases the noise increases by less than 4 dB per every 10 dB drop in the beat-note power, which is in good agreement with our previous studies on direct CLaDS [18]. The data acquisition in the field was performed by averaging 150 consecutive points (instead of 50 acquired in the laboratory test) and with a higher duty cycle (12.5% *vs.* 5% in the laboratory), which results in an effective acquisition time of 37.5 s (*vs.* 10 s in the laboratory). Therefore, to directly compare both results the field characteristics should be multiplied by a factor of (37.5/10)^1/2^ ≈ 1.936. Indeed the experimental data presented in Figure 9 show the same slope for both data sets, except the field results are shifted down by a constant value that corresponds to 1.911 times reduction in noise. This is in excellent agreement with the predicted factor of 1.936 and confirms that in both tests the system was operating within the same noise limit, indicating that no other unpredictable noise sources have been introduced in the less-controlled environment in the field.

**Figure 10.**
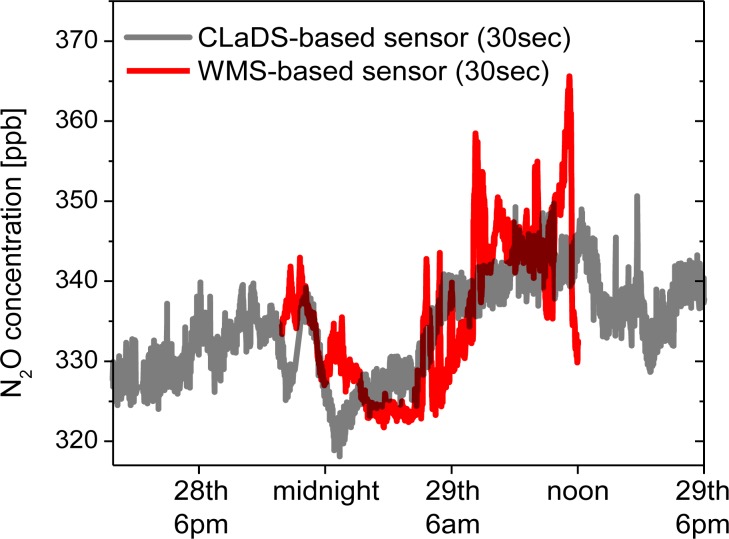
N_2_O concentration data measured using the CM-CLaDS instrument (concentration data in gray, 30 s average of data points recorded in 2 s intervals) and the WMS sensor (red, 30 s average of data points recorded in 0.1 s intervals) [32].

During the field deployment several significant precipitation events occurred (Figure 8), and each time a subsequent increase of N_2_O concentration was measured. For the three major rainfalls an increase in concentration is indicated by blue arrows. Although careful analysis of these events is beyond the scope of this work, such correlation has already been observed by other groups [29,30]. N_2_O is naturally produced in soil by the processes of nitrification and denitrification, which can be triggered by rain, causing an increase of the atmospheric N_2_O concentration after precipitation. As a validation of the instrument performance on 29 October (when one of these events occurred) we have performed a cross-comparison of the continuous N_2_O concentration measurement performed by the remote CM-CLaDS sensor with another experimental N_2_O sensing system co-located at the same measurement site. The second system was an open-path point sensor based on the WMS technique developed by Zondlo *et al.*[27,31]. It employed a DFB QCL operating around 2,203 cm^−1^ as a spectroscopic source and an open path multi-pass cell has been set-up to probe the N_2_O directly at atmospheric conditions. The N_2_O concentration data sets acquired by both instruments are shown in Figure 10.

Both sensors recorded a similar average trend in N_2_O concentration, including an increase after the precipitation event. The small short-term discrepancies shown in Figure 10 are primarily attributed to the different active optical paths used in both cases to interact with the atmosphere: long path-integrated CLaDS measurement *vs.* point sensing with WMS system. This cross-comparison result is very encouraging because the CM-CLaDS system was calibrated only once in the laboratory (one week before this test) and after four days of almost undisturbed operation an excellent agreement between the two different sensor systems has been found. This confirms the excellent long term stability and shows a promising robustness of this technology as well as its capability of unattended operation in field settings.

## 4. Conclusions and Future Outlook

In this work a transportable prototype QCL-based CM-CLaDS instrument designed for open-path sensing of atmospheric N_2_O concentration was developed. The CM-CLaDS system is based on molecular dispersion spectroscopy and provides sensitive and selective remote detection of N_2_O (1.2 ppmv×m/Hz^1/2^ at ambient conditions). The laboratory and field tests performed with this instrument confirmed unique properties of CLaDS technique *i.e.*, baseline-free nature and no dependence of CLaDS signal on received optical power with only minor effect on SNR. The prototype sensor was deployed in the field setting to perform remote monitoring of atmospheric N_2_O. With only 250 μW of optical power being emitted towards retroreflector, the CM-CLaDS system enabled detection of N_2_O concentration changes at single ppbv levels. The operation of the sensor was not interrupted despite large (>20 dB) transmission changes caused by the atmospheric water condensation and several precipitation events. A cross-comparison measurement with another N_2_O sensing instrument confirmed the consistent performance of CM-CLaDS and its capability of reliable long-term open-path trace-gas detection. It is worth noting that the CM-CLaDS 2f signal amplitude is directly proportional to molecular number density and does not require any power normalization or baseline correction. This is a significant advantage over other optical techniques such as LAS or WMS, which rely on accurate normalization with respect to the received optical power and can suffer from baseline and/or signal drifts due to laser intensity variations or photodetector nonlinearities. This particular property of CM-CLaDS assures strong immunity to changes in environmental conditions and provides better long-term stability of the system.

The performance of the present system can be improved by using optical frequency shifters with larger (∼1 GHz) frequency shift for optimum signal enhancement and better AR coatings for fringe noise suppression. Application of a more powerful QCL laser and a more sensitive photodetector can additionally improve the CLaDS SNR by increasing the carrier-to-noise ratio at the input of the FM demodulator, thus reducing demodulation noise. A long-term deployment of the CM-CLaDS system that will focus on environmental studies of N_2_O concentration, effects of precipitation on N_2_O emission, and on seasonal trends is currently being planned.
